# Study of the Enhanced Antimicrobial Effect of Cu/Chitosan Composite Materials on *Escherichia coli*

**DOI:** 10.1021/acsomega.6c03637

**Published:** 2026-07-06

**Authors:** Maria C. Sportelli, Giada Caniglia, Margherita Izzi, Rosaria A. Picca, Holger Barth, Sebastian Heber, Boris Mizaikoff, Nicola Cioffi, Christine Kranz

**Affiliations:** † Chemistry Department, University of Bari Aldo Moro, V. Orabona, 4, 70126 Bari, Italy; ‡ Institute of Analytical and Bioanalytical Chemistry, Ulm University, Albert Einstein-Allee 11, 89081 Ulm, Germany; § Institute of Experimental and Clinical Pharmacology, Toxicology, and Pharmacology of Natural Products, Ulm University Medical Center, Albert Einstein-Allee 11, 89081 Ulm, Germany

## Abstract

Here, we report the development and comprehensive characterization of copper–chitosan (Cu/CS) antimicrobial films, investigated using a combination of spectroscopic techniques and atomic force microscopy (AFM), to elucidate their chemical composition, Cu^2+^ ionic release behavior, and physicochemical properties. Particular emphasis was placed on correlating the chemical and surface properties of the films with their antimicrobial performance, to relate the effects of Cu^2+^ release and physicochemical properties of the films to the antimicrobial action. AFM was employed to analyze the morphology, surface roughness, and integrity of *Escherichia coli* bacterial cells incubated on Cu/CS films for short (2 h) and prolonged (20 h) exposure times. Time-dependent morphological changes were observed in bacteria in contact with the Cu/CS surfaces, indicating pronounced cellular stress and progressive loss of viability over exposure time. These changes included a transition from the typical rod-like morphology of healthy *E. coli* to coccoid-shaped cells, the formation of membrane bleb-like protrusions, increased cell clustering, and severe membrane disruption ending in cell lysis. The observed variations in bacterial morphology and surface characteristics provided a direct proof of damage to the bacterial envelope and reduced cellular viability. These findings strongly suggest a combined antimicrobial effect arising from the simultaneous action of Cu^2+^ ion release (220 ppb in 24 h) and the intrinsic bioactivity of the CS matrix. The results highlight the potential of Cu/CS films as effective antimicrobial materials and support their further development for applications in antimicrobial coatings, packaging, and biomedical surfaces.

## Graphical abstract

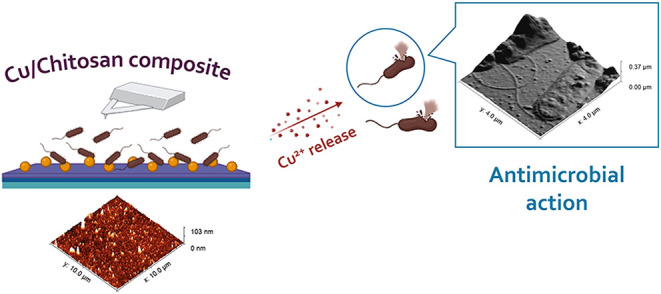

## Introduction

Antibacterial coatings play a critical role in the prevention and control of bacterial infections, predominantly in healthcare, food packaging, and biomedical device applications, where surface-related bacterial colonization represents a key route for infection transmission. These coatings can exert their antimicrobial activity either through direct contact with bacterial cells (contact-active or contact-killing mechanisms) or through the release of antimicrobial species into the surrounding environment.^1^ Multifunctional systems, such as polyhydroxyalkanoates (PHAs) combined with ZnO and sepiolite,^2^ ε-poly l-lysine with quaternary ammonium salts (QACs)^3^ or inorganic particles,^4,5^ and many other combinations of intrinsic antimicrobial polymers and antimicrobial additives,^6^ that combine contact-killing^7^ and release-based mechanisms,^8^ offer a significant advantage if compared to single-strategy coatings. In fact, they can enhance antibacterial efficacy while potentially reducing the probability of bacterial adaptation and the development of antimicrobial resistance (AMR).^9^ Such hybrid approaches are increasingly considered as a promising strategy to address the growing global challenge of AMR.

In recent years, substantial attention has been devoted to cationic polymers, such as chitosan (CS) and its derivatives, as effective contact-killing antimicrobial surfaces.^10^ CS is particularly attractive because of its biocompatibility, biodegradability, and ability to disrupt bacterial membranes through electrostatic interactions between its positively charged amino groups and the negatively charged components of bacterial cell envelopes. In parallel, the incorporation of transition metal (TM) nano- and microparticles has become a foundation stone of antimicrobial materials research.^11^ Numerous studies have demonstrated that TM particles, including zinc (Zn), copper (Cu), and silver (Ag), display broad-spectrum antimicrobial activity.^12^ Importantly, their multitarget modes of action, ranging from membrane damage and protein inactivation to oxidative stress induction, render the occurrence of bacterial resistance highly unlikely.^13,14^

Among these materials, submicrometer-sized Cu particles have recently emerged as particularly promising antimicrobial agents.^15–19^ Cu is well-known for its effective antibacterial properties, while particles with average diameters larger than 100 nm exhibit negligible nanotoxicological risks compared to smaller nanoparticles.^20^ This size regime allows for prolonged antimicrobial activity while minimizing concerns related to particle internalization and long-term cytotoxicity.^21^ In this context, we introduce a multicomponent antimicrobial coating that integrates the contact-killing properties of CS with the controlled and long-lasting release of Cu^2+^ ions from submicrometer-sized Cu particles. The combination of these two antimicrobial strategies likely enhances antibacterial performance and long-term stability, making such coatings highly relevant for the development of advanced antimicrobial surfaces.

Since 2001, Dufrêne and co-workers have pioneered the application of atomic force microscopy (AFM) to probe bacterial cells at the nanoscale under physiologically relevant conditions.^22,23^ Nowadays, AFM is established as a powerful tool to visualize and quantify the real-time effects of antimicrobial agents on living bacteria. For example, by combining high-resolution imaging with force spectroscopy, Dufrêne revealed how antibiotics and antimicrobial peptides alter cell surface architecture and mechanics.^24^ AFM can measure changes in cell wall stiffness, adhesion, and turgor pressure during antimicrobial challenge, also in the presence of antimicrobial ions (such as Zn^2+^).^25^ This approach was recently used by Caniglia et al.^26^ to study the antimicrobial activity of Ag microspots against *Escherichia coli*, using AFM-based force spectroscopy to analyze bacterial adhesion and cell elasticity. They observed that the bacterial outer membrane experienced remarkable structural changes when in the vicinity of Ag spots. Indeed, bacteria showed increased hydrophilicity and loss of stiffness. These measurements granted direct biophysical proof of drug-induced damage at the single-cell level. Hence, AFM allows bridging molecular interactions and phenotypic responses by correlating nanoscale forces with antimicrobial mechanisms of action.^27^ Single-molecule AFM has been used to map antibiotic–cell surface interactions with unprecedented specificity.^28,29^ This work enabled quantitative analysis of bacterial heterogeneity in antimicrobial susceptibility.

Furthermore, AFM has been extensively employed to characterize the antimicrobial activity of composite and multifunctional coatings, revealing morphological and mechanical changes at the single-cell level with high lateral resolution.^26,30–32^ The ability to image intact bacterial cells across macroscopic surfaces enables the assessment of population heterogeneity and the identification of localized damage induced by antimicrobial interfaces.^33^ AFM can be operated in liquid, thereby preserving bacterial native structure and enabling time-resolved studies of antimicrobial interactions.^34^ AFM has been successfully used in studying bacteria and their behavior depending on substrate and environment.^35^ The pioneering studies on this topic were those of Bremer et al.^75^ (involving Cu) and Steele et al. (using stainless steel).^36^ Also, steel, aluminum, rubber, and polypropylene have been studied as substrates.^37^ These studies allowed understanding that the shape and composition of the substrate influence the morphology, physiology, and adhesion of bacterial cells.^36,38,39^

In this work, we present a spectroscopic and AFM study on multicomponent Cu/CS antimicrobial films. The aim was to elucidate the relationship between bacterial morphological changes and the structure and composition of the bioactive surfaces. Although the antimicrobial activity of Cu/CS is well-known and documented in the literature,^40–42^ AFM is here employed to probe changes in bacterial morphology, surface roughness, and circularity, providing high-resolution, single-cell-level evidence of antimicrobial mechanisms.

These Cu/CS materials are enhanced, eco-friendly packaging and biomedical solutions. As a natural, biodegradable material, CS-based films offer an environmentally friendly alternative to synthetic plastics, with Cu being a safe oligoelement. In addition, the incorporation of Cu particles into the CS matrix provides strong antimicrobial properties. Specifically, the effects of CS and Cu/CS composite coatings were investigated by using *E. coli* as a model Gram-negative bacterium. AFM topography and morphological analyses were performed after short-term (2 h) and long-term (20 h) incubation periods to capture both early-stage interactions and prolonged antimicrobial effects. Distinct changes in bacterial morphology, including alterations in cell shape, surface roughness, and structural integrity, were identified as a function of the contact time and the presence or absence of Cu particles within the coating. Bacterial morphological damage does not necessarily indicate a loss of viability. Accordingly, cell viability was correlated to the morphological alterations observed by AFM, demonstrating the antimicrobial activity of the composite coating. These findings highlight the importance of antimicrobial surface design and demonstrate the utility of AFM as a powerful tool for correlating surface properties to bacterial responses at the single-cell level.

## Experimental Section

### Materials

Polyvinylpyrrolidone (PVP, 15K, MW ∼ 10.000) was acquired from Fluka. d­(+)-Glucose (≥99.5%, ACS reagent), copper sulfate pentahydrate (CuSO_4_·5H_2_O, 99.995% trace metals basis), sodium hydroxide (NaOH, pellets, 98%, ACS reagent), ethanol (EtOH, absolute, for HPLC, ≥99.8%), nitric acid (HNO_3_, 67%, Trace-SELECT Ultra, for ultratrace analysis), sodium chloride (NaCl, Trace-SELECT Ultra, ≥99.999%, for ultratrace analysis), sodium phosphate monobasic (NaH_2_PO_4_, Trace-SELECT, ≥99.99%, for trace analysis), and sodium phosphate dibasic (Na_2_HPO_4_, Trace-SELECT, ≥99.99%, for trace analysis) were purchased from Merk Sigma-Aldrich. Chitosan (CS, hydrochloride, 100%, maximum total heavy metal content <40 ppm, for biological agriculture) was obtained from Agrilæte. Acetic acid (HAc, glacial, for HPLC) was from Baker Analyzed. All reagents were employed as received. Milli-Q water was used throughout the experiments. N^+^/Sb-doped Si wafers were purchased from Si-Mat Silicon Materials, and cut into 2 × 2 cm^2^ squares. They were washed by subsequent sonication in water and EtOH.

### Preparation of Cu Particles and Composite Thin Films

Preparation of Cu particles stabilized by PVP (Cu@PVP) has been described elsewhere.^20,43^ Briefly, two equal volumes of glucose 0.08 g/mL and CuSO_4_ 0.01 M were mixed at 80 °C. Both solutions were prepared using 2 mM PVP as the solvent. The pH value was adjusted to ∼9 with 5% NaOH for a 2 h synthesis. The colloid was then washed with water and acetone, and the resulting powder was used for the preparation of composite thin films. First, we prepared a 1.5%_w/w_ CS solution in 1%_v/v_ acetic acid. After dissolving the biopolymer, we added Cu@PVP particles suspended in EtOH, at a final concentration in the composite of 1 g/L. After mixing at 65 °C for 30 min, the obtained solution was then used for spin coating on Si substrates (500 μL on 2 × 2 cm^2^ substrates). Bare CS films were prepared accordingly, without adding Cu@PVP particle suspension. We used a two-step spin process with the following program: 30″ at 500 rpm, and 60″ at 2000 rpm.

### Characterization of CS and Cu/CS Composite Thin Films

X-ray photoelectron spectroscopy (XPS) measurements were performed using a PHI Versaprobe II spectrometer, with monochromatized Al–Kα radiation (1486.6 eV). Experimental details and data treatment protocols for elemental analysis and peak fitting are described elsewhere.^44^ The binding energy (BE) scale was calibrated taking the C1s spectrum at BE = 284.8 eV (aliphatic component) as reference. Cumulative spectroscopic quantification of Cu^2+^ ions released into the aqueous solution was performed using electrothermal atomic absorption spectroscopy (ETAAS). The Cu/CS samples were placed in contact with 1 mL of phosphate buffer saline solution (pH = 6.8, *I* = 0.1) for up to 24 h. Aliquots of 200 μL were analyzed after mineralization with 0.2%_v/v_ HNO_3_, with a PerkinElmer PinnAAcle AS 900Z double-beam spectrometer. Data analysis and processing were performed by OriginPro 2021 software, version 9.8.0.200 (Origin Lab Corporation). Topography images of CS and Cu/CS thin film surfaces were acquired using a Keysight AFM system Model 5500. AFM images were obtained in contact mode, in air, using pyramidal Si_3_N_4_ probes with a typical tip radius of 15 nm (OTR8-10 Bruker AFM probes). AFM images of CS and Cu/CS thin films were analyzed using the software Gwyddion 2.58.

### Morphological Characterization of *E. coli* Incubated on CS and Cu/CS Thin Films

*E. coli* DH5-α (obtained from Clontech Laboratories, Inc., Heidelberg, Germany) was inoculated in aerobic conditions to 100 mL of 25 g/L sterile LB medium at (37 ± 1)°C overnight, using a shaking incubator (KS 4000ic control, Keison Products, U.K.). The bacterial suspension was harvested and resuspended in dilute LB medium (0.5 g/L) and incubated at (37 ± 1)°C up to a concentration of 10^6^ CFU/mL (OD_600_ = 0.6). The OD_600_ was monitored using a UV–vis spectrometer (Thermo Scientific NanoDrop One, Massachusetts, USA). The bacterial culture was then seeded on the CS and Cu/CS modified substrates by immersing them in the bacterial solution and incubating at (37 ± 1)°C for 2 or 20 h. Bare, clean Si slides were used as control samples. A 5500 AFM system from Keysight (Keysight Technologies, AZ, USA) was used for all AFM measurements. AFM images were recorded in air, in contact mode, using Si_3_N_4_ probes (MSNL-10 Bruker AFM probes; nominal spring constant of 0.1 N/m) and a scan speed of 0.52 lines/s. Although AFM imaging performed in air and in contact mode may eventually introduce artifacts (partial dehydration and mechanical deformation of bacterial cells), these imaging conditions were selected to ensure stable topographical characterization of bacterial surfaces. AFM images of bacterial cells were analyzed using the MoutainSPIP v. 9 software (Digital Surf, France). First-order leveling and flattening were applied to all images. Bacteria shape factor (circularity C ) and root-mean-square surface roughness (*R*_q_) were calculated (sample size: ∼100 nm) using ImageJ 1.53e software, with adequate adjustment of the image threshold (sample size: ∼100 nm). Statistical analyses are based on data reported as the mean of the replicates ± standard deviation. Data were subjected to ANOVA (one-way analysis of variance), *p* ≤ 0.05. Data evaluation was performed using OriginPro 2021 software, version 9.8.0.200 (Origin Lab Corporation).

### Viability Assays

A single colony of DH5-α *E. coli* was inoculated in 5 mL of 25 g/L sterile LB medium and incubated at 180 rpm at 37 °C overnight. A 50 mL culture was inoculated with the overnight culture and grown under the same conditions until an OD_600_ = 0.6 was reached. The bacterial suspension was harvested by centrifugation, and the pellet was resuspended in the same volume of diluted LB medium (0.5 g/L). *E. coli* were incubated at 37 °C in the presence or absence of the coated Si in a 6 well plate (TPP, Trasdingen, Switzerland) for the indicated times (2 h and 20 h) in a volume of 3 mL/well. For viability assays, 100 μL were transferred after the respective times in triplicate to a 96 well plate (Thermo Scientific, Massachusetts, USA) and 10 μL MTS reagent was added (CellTiter 96 AQ_ueous_ One Solution Cell Proliferation Assay (MTS), Promega, Madison, Wisconsin, USA). The conversion of MTS to a formazan dye was measured in a Tecan Infinite M1000 PRO plate reader at an absorbance of 490 nm. Cell viability was normalized to the wells, which only included *E. coli*. Statistical analysis was performed using ordinary one-way ANOVA with Šídák’s multiple comparisons test as indicated with *p* ≤ 0.05. Data evaluation was performed using GraphPad Prism version 10.6.1. (GraphPad Software). Data were plotted with OriginPro 2021 software, version 9.8.0.200 (Origin Lab Corporation).

## Results and Discussion

### X-ray Photoelectron Spectroscopy Characterization of CS and Cu/CS Thin Films and Kinetics of Cu^2+^ Ion Release

Spin-coated CS films, with and without Cu particles, were characterized by XPS. The surface composition is reported in Table 1. Besides the expected C, N, O (and Cu in the case of Cu/CS), traces of Na and Cl are appreciable, due to impurities present in the polymeric matrix. Si arises from the film substrate used for spin coating. Comparing the C1s high-resolution regions (HRRs) (Figure 1a,b), the same chemical environments can be found on CS and Cu/CS films (Table 2). The peak centered at 286.6 ± 0.2 eV is relevant to the superimposed/unresolved C–C=O, C–O, and C–N chemical environments; its relative abundance increases in the composite film (compared to bare CS films), due to the presence of PVP stabilizer, surrounding particles.^20^ A small, but appreciable Cu surface availability is demonstrated for the composite films. The Cu2p_3/2_ HRR (Figure 1c) reveals a single, asymmetric component at 932.3 ± 0.2 eV. This peak position is not enough to univocally assess the Cu chemical speciation, as the main Auger transition was not sufficiently intense to be acquired.

**Figure 1:**
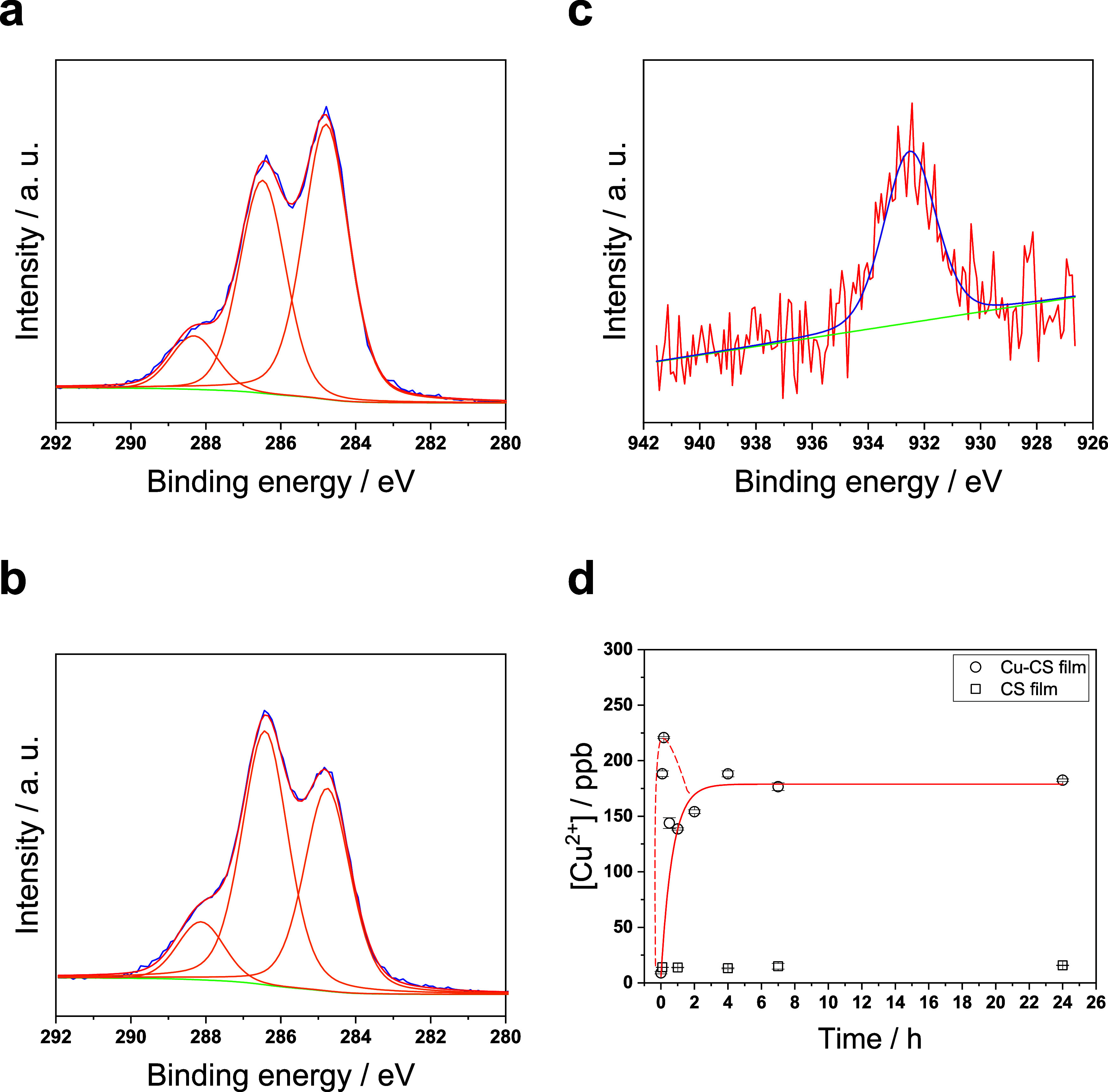
C1s high-resolution regions (HRRs) for CS (a) and Cu–CS thin films (b). Cu2p_3/2_ HRR for Cu–CS thin films (c). Kinetics of ionic release from CS and Cu–CS films over 24 h (*n* = 3) (d).

**Table 1:** Surface Atomic % of CS and Cu/CS Thin Films^a^

element	CS films	Cu/CS films
C	69.5 ± 0.5	66.1 ± 0.5
N	3.6 ± 0.5	4.6 ± 0.5
O	24.2 ± 0.5	26.3 ± 0.5
Na	<0.5	<0.5
Si	0.5 ± 0.5	0.5 ± 0.5
Cl	2.2 ± 0.5	2.4 ± 0.5
Cu	/	0.3 ± 0.2

a Error is expressed as the larger value between the error associated with a single quantification and one standard deviation, calculated on at least three replicate analyses. Na impurities related to the polymer are detectable. Si is related to the substrate.

**Table 2:** CS and Cu/CS C1s Signal Components^a^

CS film			Cu/CS films		
Position (eV)	Attribution	Relative At%	Position (eV)	Attribution	Relative At%
284.8 ± 0.1	C–C, C–H	49.4 ± 0.5	284.8 ± 0.1	C–C, C–H	43.5 ± 0.5
286.4 ± 0.2	C–C=O, C-O, C-N	40.4 ± 0.5	286.4 ± 0.2	C–C=O, C-O, C-N	46.6 ± 0.5
288.4 ± 0.2	C=O	10.2 ± 0.5	288.4 ± 0.2	C=O	9.9 ± 0.5

a Error is expressed as the larger value between the error associated with a single quantification and one standard deviation, calculated on at least three replicate analyses.

However, based on data from the literature on PVP-capped Cu particles, we assume that Cu is mainly present in the Cu(0) oxidation state.^20^ Cu/CS thin films were exposed to the phosphate-buffered saline (PBS) solution at pH 6.8, mimicking physiological conditions for bacterial growth. The obtained release kinetics, over a 24 h time span, are reported in Figure 1d.

A release trend already observed for similar composites,^20,43,45^ relevant to pseudo-first-order kinetics, was found. The [Cu^2+^] concentration in solution reached 175 ± 25 ppb at the *plateau*, with a kinetic release constant of 3.8 ± 0.9 h^–1^. The highest released [Cu^2+^] concentration, due to Cu^2+^ supersaturation in solution,^46^ that equals 220.9 ± 0.8 ppb, was lower than the highest one reported to be human-safe in food products by the European Food Safety Agency (EFSA).^47^ A negligible and almost constant release below 5 ppb was found for bare CS samples. Being the Cu particles embedded within the polymer matrix (as evidenced by XPS and AFM characterizations, *vide infra*), the release of entire Cu particles in the contact solution appears unlikely.

Indeed, the dissolution of cupric ions from particles happens because the Cu surface undergoes oxidation in the presence of oxygen, etc., since oxygen acts as an electron acceptor and chlorides promote CuO dissolution as cupric chlorocomplexes.^48^ Cu^2+^ ions can penetrate membranes, catalyze the formation of reactive oxygen species (ROS), disrupt enzymes, and damage DNA.

### Morphological Characterization of Cu/CS Thin Films

The surface morphology was analyzed using an AFM. The AFM images are depicted in Figure 2.

**Figure 2:**
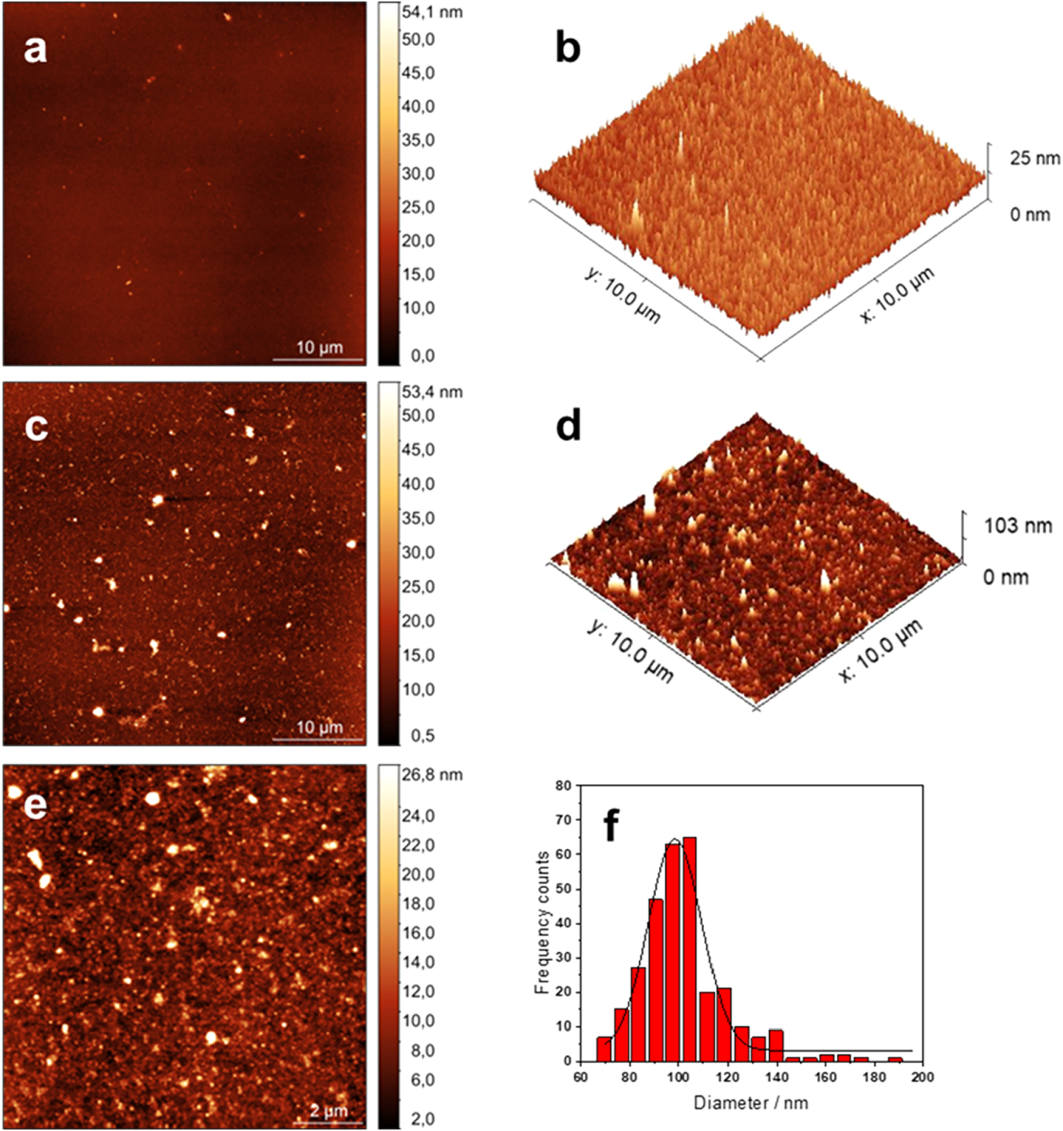
AFM micrographs of CS (a, b) and Cu/CS (c–e) thin films. Average diameter of Cu particles as measured from the micrograph (e).

The CS film shows a smooth and homogeneous surface (Figure 2a,b), with a root-mean-square roughness (*R*_q_) of 2.4 ± 0.8 nm. Conversely, Cu/CS films (Figure 2c–e) were characterized by the presence of spheroidal structures on the surface, due to the presence of Cu particles in the polymer matrix. The *R*_q_ was determined as 10.3 ± 0.4 nm. The average size distribution of the Cu particles was determined with an average diameter of 100 ± 20 nm (Figure 2f). This value corresponds to about half of the value evaluated for bare Cu particles,^43^ thus indicating that particles are probably half-buried in the polymer matrix.

### Morphological Characterization of *E. coli* Incubated on Thin Films for 2 h

Strains of *E. coli* DH5-α at the end of the exponential growth phase were diluted to a concentration of 10^6^ CFU/mL in culture medium and then incubated on Si substrates modified with CS and Cu/CS thin films. A bare Si substrate was used as a control sample. After drying, AFM images of bacteria incubated for 2 h were recorded; images are shown in Figure 3. *E. coli* cells seeded on bare Si exhibited an intact rod-shaped morphology with no visible ruptures of the cell membrane (Figure 3a–c). The irregular structures observed around the bacterial cells were attributed to residual salt deposits. The measured cell dimensions are consistent with values reported in the literature.^49^ In contrast, incubation of bacteria on CS-coated substrates (Figure 3d–f) indicated some cell damage, ranging from grooves and lesions in the outer membrane (particularly at the apical edges) (Figure 3e), to a wrinkled bacterial surface (Figure 3f). These effects can be attributed to electrostatic interactions between CS, a cationic polymer, and the anionic phospholipid moieties of the bacterial membrane under physiological conditions, which are predominantly located at the apical regions (Figure S1). Such interactions likely initiate cell wall damage.^10^ Exposure of *E. coli* to Cu/CS-coated substrates caused more pronounced morphological alterations. After 2 h of incubation on Cu/CS, the bacterial surfaces appeared distorted (Figure 3g,h), and cells exhibiting partial loss of cytoplasmic material were observed^50^ (Figure 3i). The films containing Cu particles can release antimicrobial Cu^2+^ ions in a controlled and long-lasting way (refer to Figure 1).

**Figure 3:**
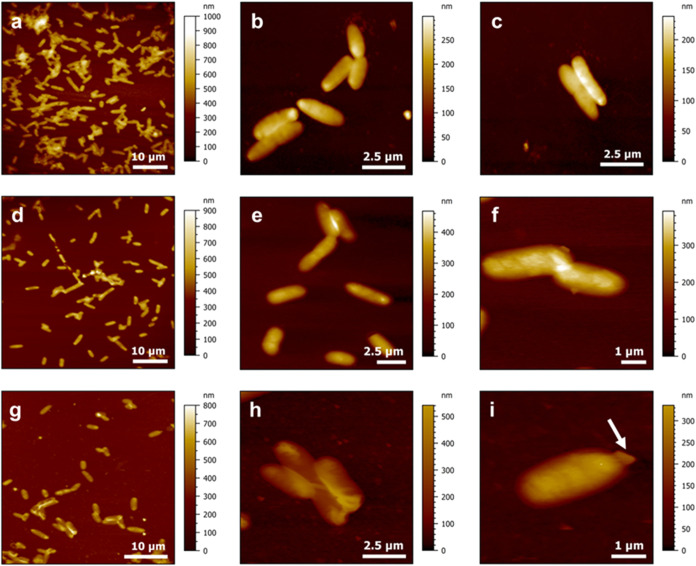
AFM images of *E. coli*, after 2 h of incubation on bare Si as the control sample (a–c), CS-coated Si (d–f), and Cu/CS-coated Si (g–i). Partial loss of cytoplasmic material is highlighted in panel (i).

The combined electrostatic interactions between the cationic polymer and Cu^2+^ ions, with the negatively charged bacterial membrane, compromise its structural integrity, inducing mechanical stress and eventual rupture (Figure 3h).^51^ Apical and edge collapse was particularly evident in this sample (Figure S2), further supporting the combined effects of CS and Cu^2+^ ions on membrane destabilization.^52^

A major proposed pathway involving the release of Cu^2+^ ions over short time spans foresees the generation of ROS, including hydroxyl radicals and superoxide species, through redox cycling between Cu^+^ and Cu^2+^. These ROS induce oxidative stress, lead to lipid peroxidation of the bacterial envelope, and oxidize membrane proteins, ending in damage to nucleic acids and intracellular enzymes.^53,54^ The resulting loss of membrane integrity disrupts ionic homeostasis and cellular respiration, ultimately causing cell death.^55^ Through AFM, these phenomena are visible from leakage of intracellular material, leading to partial flattening of the cells (Figure 3h,i). These findings support the contact-killing mechanism mediated by ROS and also direct membrane disruption from cations.^56–58^

### Morphological Characterization of *E. coli* Incubated on Thin Films for 20 h

As expected, prolonged incubation up to 20 h resulted in a markedly enhanced bacterial damage. The Si control sample (Figure 4a–c) exhibited clear signs of viable bacterial growth (considering that the same amount of bacteria was seeded for both the 2 and 20 h experiments), with a dense layer of *E. coli* cells covering the surface. Some cells appeared piled-up (Figure 4c), likely indicating the early stages of biofilm formation.^59^ Exposure to the CS-coated substrate (Figure 4d,e) caused partial cell lysis and the appearance of “cellular ghosts” (cells that had lost portions of their cytoplasmic content),^50^ as shown in Figure 4f. These effects arise from irreversible disorganization of the cell membrane and subsequent cytoplasmic leakage, induced by prolonged electrostatic interactions between the bacterial envelope and the cationic CS polymer (Figure S3a,b).^9,60^

**Figure 4:**
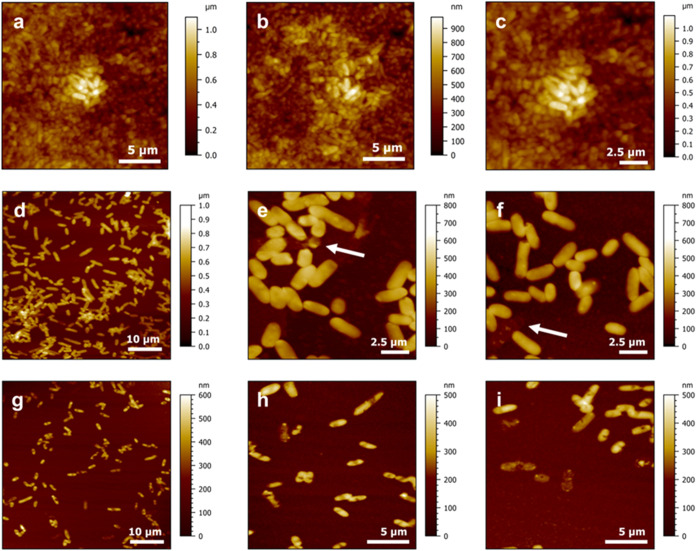
AFM images of *E. coli*, after 20 h of incubation on bare Si as the control sample (a–c), CS-coated Si (d–f), and Cu/CS-coated Si (g–i). Cellular ghosts are highlighted in panels (e, f).

Following 20 h of incubation on the Cu/CS substrate, even more pronounced structural alterations were observed. Many bacteria appeared as “ghosts,” while the remaining intact cells displayed a corrugated morphology (Figure S4a). Numerous bleb-like protrusions extend from cell surfaces. These protrusions (Figure S4b) are attributed in the literature to the remains of the outer membrane and peptidoglycan layer after exposure to antimicrobial agents,^61^ like CS and Cu^2+^ ions released from the surface upon incubation.^62^ This last phenomenon can be univocally attributed to local peptide alterations caused by ROS production. Prolonged ionic release, moreover, can lead to significant variations in membrane permeability and osmotic imbalance, still leading to increased presence of protrusions and blebs,^63,64^ as well as increased surface roughness (*vide infra*, Figure 7).

### Statistical Evaluation of Viability, Topological Parameters, and Shape Factors of *E. coli*

Bacterial morphological damage alone does not necessarily imply a loss of viability and, therefore, cannot, by itself, substantiate antimicrobial efficacy. To address this limitation, viability measurements were used to complement the morphological observations described above. Specifically, the effects observed on bacterial structure were validated by assessing the viability of *E. coli* following incubation in the absence or presence of CS- and Cu/CS-coated Si substrates (Figure 5).

**Figure 5:**
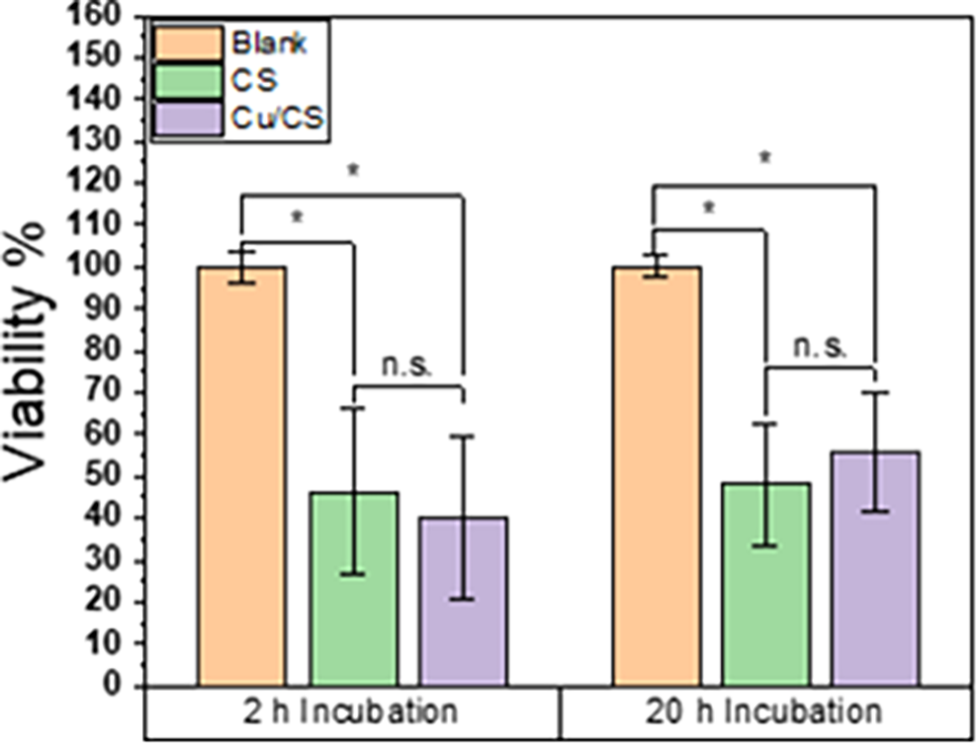
Viability of *E. coli* incubated in the absence or presence of either CS or Cu/CS-coated Si for 2 and 20 h (9 total measurements, from three independent experiments). Viability was measured by the MTS assay. Data were reported as mean value ± st. deviation; statistical significance is denoted by *p < 0.05 (n.s. = not significant).

A statistically significant reduction of viability was shown for CS and Cu/CS coatings for both contact times. Taken together, the viability was significantly reduced when *E. coli* were incubated either in the presence of CS or Cu/CS-coated Si. Interestingly, the maximum effect on viability was already observed after an incubation period of 2 h.

When exposed to external stress factors, *E. coli* is known to change its phenotype from elliptical to quasi-spherical, to maintain its cell wall integrity, increasing its hydrophobicity, and decreasing its adhesion to charged surfaces (like the used CS and Cu/CS films).^34^ When this happens, division-regulating proteins are overexpressed.^65^ Such behavior is typical of the exposure to sub-MIC (i.e., subminimum inhibitory concentration) of antimicrobials in the culture medium. An indicative shape factor, i.e., the circularity, was measured on ∼100 bacterial cells, after 2 and 20 h of incubation (Figure S5), and statistically evaluated by applying an ANOVA test (Figure 6).^66^ Means and standard deviations were retrieved after Gaussian fitting of C parameter distributions (Figure S5). The dimensions of the error bars are due to the intrinsic variability of bacterial samples.

**Figure 6:**
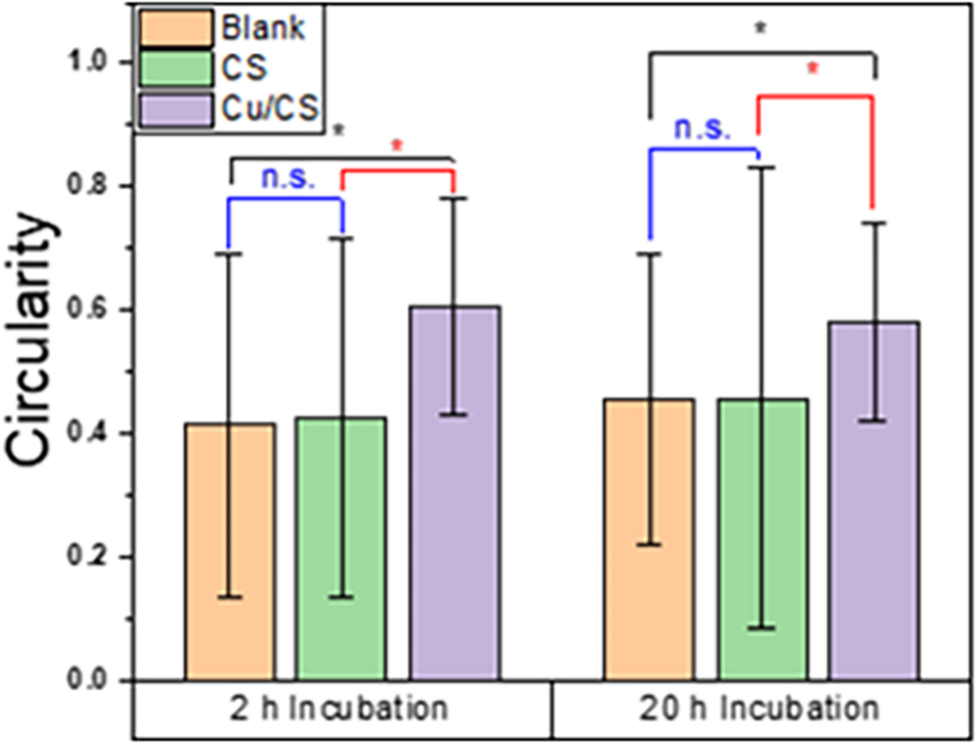
Evaluation of circularity C for *E. coli* samples incubated for 2 h and 20 h (∼100 bacterial cells, error bars correspond to the st. deviation of three different samples). Data were reported as mean value ± st. deviation; statistical significance is denoted by *p < 0.05 (n.s. = not significant).

Values of circularity ( C, i.e., roundness of an object) near 1.0 define coccoid-shaped cells, while values of circularity below 0.7 are more characteristic of rod-shaped bacteria. Statistical analysis of the values reported in Figure 6 revealed no significant difference between control samples and *E. coli* incubated on CS substrates.

This was true for both 2 h and 20 h incubation times. Indeed, significant differences were observed for samples incubated on Cu/CS substrates with respect to control and CS-incubated ones, despite the contact time.

Even upon short incubation time, the presence of Cu on the substrate transforms the shape of the bacterial cells toward a coccoid-like shape, i.e., toward a reduction in cell length. This is as an effect of the sub-MIC release of Cu^2+^ ions from the surface.^67^ Alteration of cell phenotype could then be attributed mostly to the release of Cu^2+^ ions. Alteration of bacterial shapes can affect cell packing and van der Waals interactions. Transforming into the coccoid-like shape reduces the probability of undergoing the effect of external stress.^68^ We also correlated the contact time with the cell surface roughness, for the different substrates, as it is linked to the extent of the cellular damage (Figure 7a). In general, the roughness of the outer membrane increases with the increase in membrane damage.^49^ We determined the root-mean square roughness values (*R*_q_) of ∼100 individual bacteria for each sample, and for two incubation times. Results are shown in Figure 7a. Lowest *R*_q_ values were registered, for both incubation times, in the case of control samples: 22 ± 4 nm (*n* = 3) for 2 h incubation, and 25 ± 4 nm (*n* = 3) for 20 h.^69^ These values are not significantly different. For the 2 h incubation, *R*_q_ increased significantly (with respect to control samples) for bacteria in contact with CS substrates, reaching 31 ± 6 nm after 2 h and 44 ± 13 nm after 20 h. An *R*_q_ value of 51 ± 14 nm was registered for bacteria incubated on Cu/CS for 2 h and of 54 ± 7 nm after 20 h.^34^ The latter values did not reveal to be statistically different between 2 h and 20 h incubation. The statistical analysis of the surface roughness values corroborated well with the morphological observations made on *E. coli* after incubation on antimicrobial surfaces, as also reported in the literature for coatings containing Ag nanoparticles.^70,71^ Considering the data presented in Figures 3 and 4, it is evident that the density of bacteria (i.e., the abundance of bacteria within the analyzed surface area) on the different films (as a function of the incubation time) is different. Hence, surface coverage % was statistically evaluated on 40 × 40 μm^2^ frames (Figure 7b). Coverage on the control samples was dramatically different between 2 h and 20 h: the absence of any antimicrobial led to a massive bacterial cell growth over time, passing from 18 ± 3 to 76 ± 5%. When in contact with the CS substrate, coverage % was again significantly different; the smaller difference in coverage % between 2 and 20 h is attributed to the electrostatic interaction between positively charged CS and negatively charged *E. coli*, which promotes certain bacterial damage over time.^72^ Interestingly, no significant difference was appreciable on Cu/CS substrates: this can be attributed to the significant bacteriostatic effect exerted by the Cu^2+^ ion release and the possible enhanced effect of Cu and CS.^73,74^

**Figure 7:**
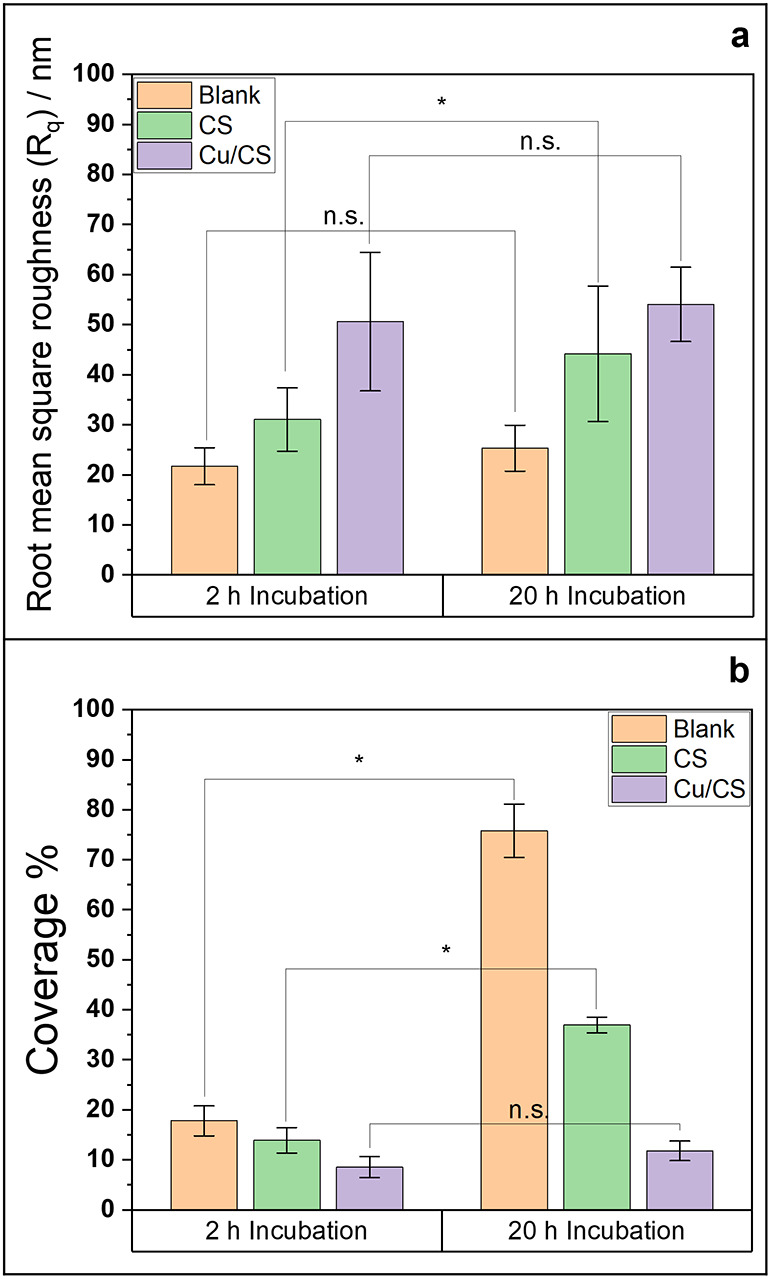
(a) Surface roughness values of *E. coli* cells, determined on ∼100 individual cells, after incubation on bare Si (control sample, orange), CS-coated Si (green), and Cu/CS-coated Si (purple). Data were reported as mean value ± st. deviation; statistical significance is denoted by *p < 0.05 (n.s. = not significant). (b) Bar chart of the measured coverage (error bars correspond to st. deviation of three different samples). Statistical significance is denoted by *p < 0.05 (n.s. = not significant).

## Conclusions

In this study, we prepared and characterized antimicrobial thin films based on a natural polymer (chitosan) and submicrometer Cu particles. Thin films were characterized in terms of surface composition, surface topography, and kinetics of ionic release. They also revealed to have antimicrobial efficacy against *E. coli* (as a model microorganism). This paper provides a visual demonstration of the actual effect of combined antimicrobial films on living bacterial cells as a function of time. Microscopic results were corroborated by viability assays, which demonstrated that bacterial morphological changes also corresponded to a loss of viability. The results from our AFM studies proved that it is possible to distinguish between the antimicrobial action of CS films (mainly exerted by contact and electrostatic interaction) and the improved one of Cu/CS films, where the presence of Cu^2+^ ions yields an orthogonal antimicrobial effect that potentiates the action of CS.

## Supplementary Material



## Data Availability

The data underlying this study are available in the published article and its Supporting Information.

## Notes

AFM images of *E. coli* incubated on CS for 2 h. Details of partially collapsed bacteria (a). Details of grooves and flagella for a bacterium incubated on CS for 2 h (b) (Figure S1); AFM images of *E. coli* incubated on Cu/CS for 2 h. Details of bacteria with cell edges collapse (a, b). Details of the bacterial surface showing apical collapse after incubation on Cu/CS for 2 h (b) (Figure S2); AFM images of *E. coli* incubated on CS for 20 h. Details of lysed bacteria (a, b) (Figure S3); AFM micrographs of *E. coli* incubated on Cu/CS for 20 h. Details of cellular ghosts (a). Details of bleb-like protrusions after incubation on Cu/CS for 20 h (b) (Figure S4); evaluation of circularity of *E. coli* incubated for 2 h (top) and 20 h (bottom). Control sample (left), *E. coli* incubated on CS (center), *E. coli* incubated on Cu/CS (right) (Figure S5) (PDF)
