# Supplementary material for: Study of the Enhanced Antimicrobial Effect of Cu/Chitosan Composite Materials on Escherichia coli
Source: ACS Omega. 2026 Jul 6;11(28):42546–56. doi: 10.1021/acsomega.6c03637 (PMC13393368; doi:10.1021/acsomega.6c03637)
Supplement: Supplementary file 1 [file ao6c03637_si_001.pdf]

Supplementary Material

Study of the enhanced antimicrobial effect of Cu/Chitosan composite materials on *Escherichia coli*

Maria. C. Sportelli[1], Giada Caniglia[2], Margherita Izzi[1], Rosaria A. Picca[1], Holger Barth[3], Sebastian Heber[3], Boris Mizaikoff[2], Nicola Cioffi[1], and Christine Kranz[2*]*

[1] Chemistry Department, University of Bari Aldo Moro, V. Orabona, 4, 70126, Bari, Italy.

[2] Institute of Analytical and Bioanalytical Chemistry, Ulm University, Albert Einstein-Allee 11, 89081 Ulm, Germany.

[3] Institute of Experimental and Clinical Pharmacology, Toxicology, and Pharmacology of Natural Products, Ulm University Medical Center, Albert Einstein-Allee 11 - 89081 Ulm, Germany.

[*] Correspondence: christine.kranz@uni-ulm.de; maria.sportelli@uniba.it

KEYWORDS: Antimicrobial coatings; Copper; Chitosan; *Escherichia coli*; Atomic force microscopy; Cell roughness; Cell morphology.

<u>Micrographs of *E. coli* incubated on CS and Cu/CS</u>

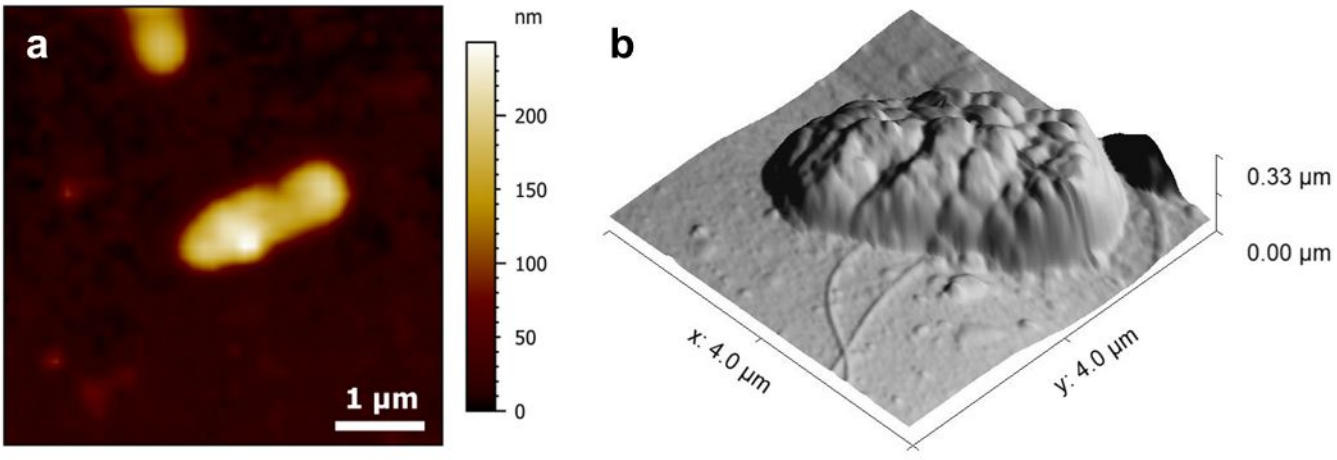

Figure S1. AFM images of *E. coli* incubated on CS for 2 h. Details of partially collapsed bacteria (a). Details of grooves and flagella for a bacterium incubated on CS for 2 h (b).

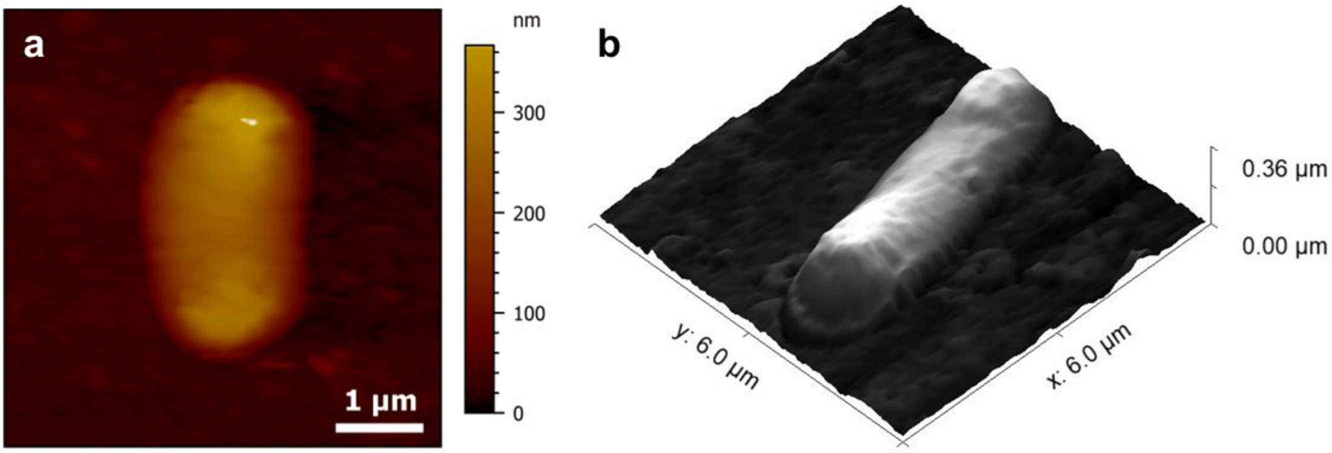

Figure S2. AFM images of *E. coli* incubated on Cu/CS for 2 h. Details of bacteria with cell edges collapse (a, b). Detail of the bacterial surface showing apical collapse after incubation on Cu/CS for 2 h (b).

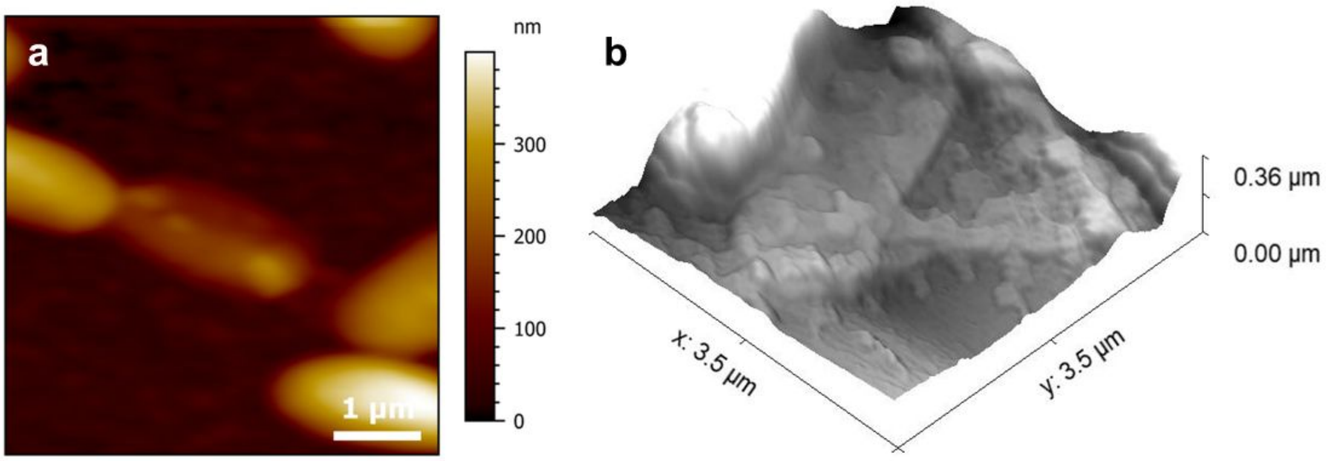

Figure S3. AFM images of *E. coli* incubated on CS for 20 h. Details of lysed bacteria (a, b).

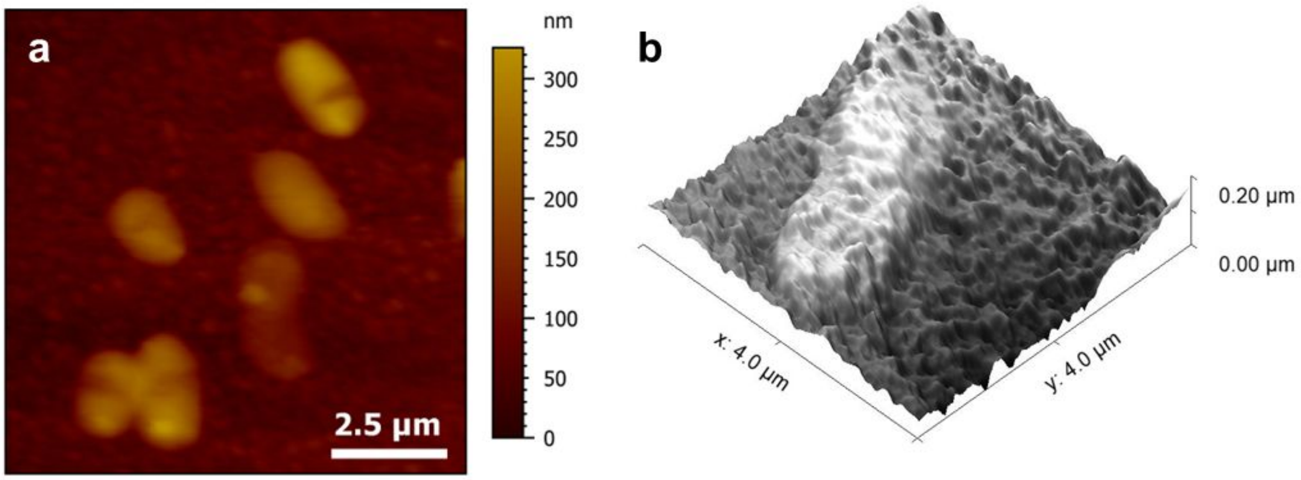

Figure S4. AFM micrographs of *E. coli* incubated on Cu/CS for 20 h. Details of cellular ghosts (a). Details of bleb-like protrusions after incubation on Cu/CS for 20 h (b).

Evaluation of Circularity (ℂ) and Gaussian curve-fit of ℂ distributions.

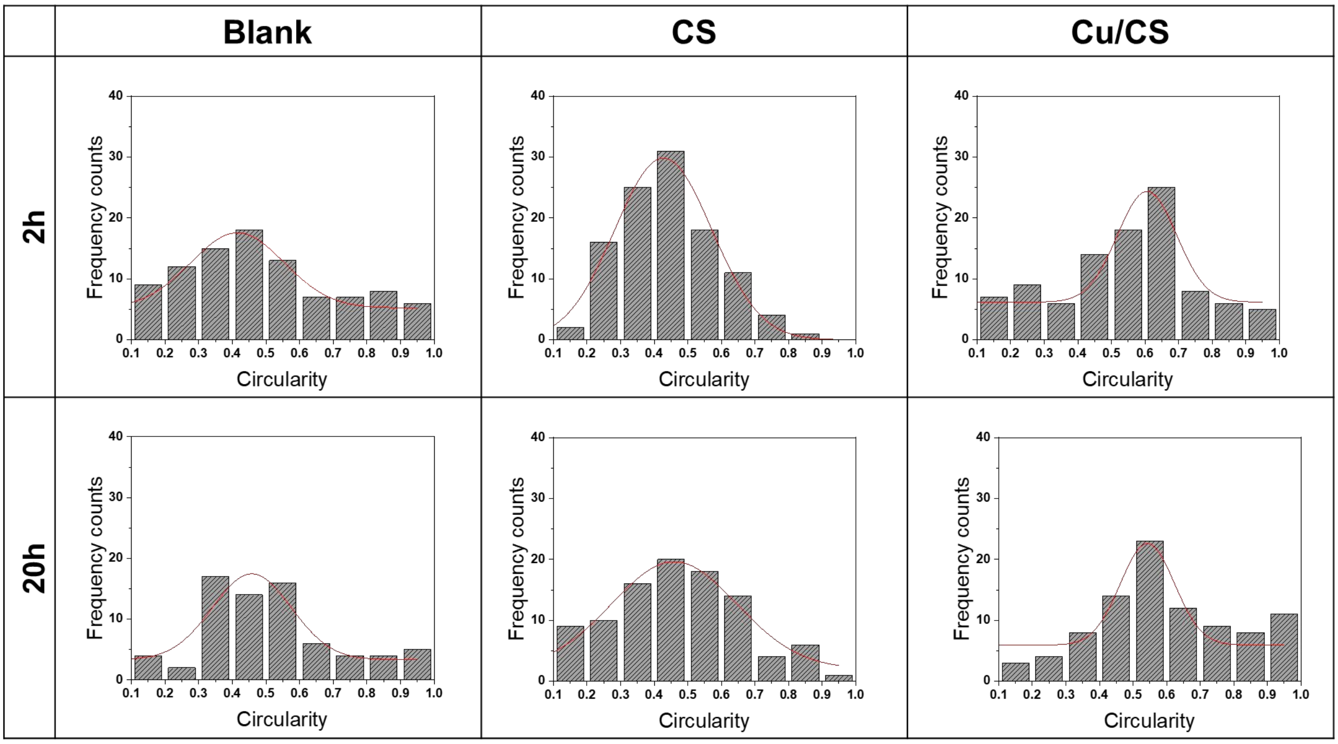

Figure S5. Evaluation of circularity of *E. coli* incubated for 2 h (top) and 20 h (bottom). Control sample (left), E. coli incubated on CS (center), E. coli incubated on Cu/CS (right).
